# Quality control of B-lines analysis in stress Echo 2020

**DOI:** 10.1186/s12947-018-0138-7

**Published:** 2018-09-25

**Authors:** Maria Chiara Scali, Quirino Ciampi, Eugenio Picano, Eduardo Bossone, Francesco Ferrara, Rodolfo Citro, Paolo Colonna, Marco Fabio Costantino, Lauro Cortigiani, Antonello D’. Andrea, Sergio Severino, Claudio Dodi, Nicola Gaibazzi, Maurizio Galderisi, Andrea Barbieri, Ines Monte, Fabio Mori, Barbara Reisenhofer, Federica Re, Fausto Rigo, Paolo Trambaiolo, Miguel Amor, Jorge Lowenstein, Pablo Martin Merlo, Clarissa Borguezan Daros, José Luis de Castro e Silva Pretto, Marcelo Haertel Miglioranza, Marco A. R. Torres, Clarissa Carmona de Azevedo Bellagamba, Daniel Quesada Chaves, Iana Simova, Albert Varga, Jelena Čelutkienė, Jaroslaw D. Kasprzak, Karina Wierzbowska-Drabik, Piotr Lipiec, Paulina Weiner-Mik, Eva Szymczyk, Katarzyna Wdowiak-Okrojek, Ana Djordjevic-Dikic, Milica Dekleva, Ivan Stankovic, Aleksandar N. Neskovic, Angela Zagatina, Giovanni Di Salvo, Julio E. Perez, Ana Cristina Camarozano, Anca Irina Corciu, Alla Boshchenko, Fabio Lattanzi, Carlos Cotrim, Paula Fazendas, Maciej Haberka, Bozena Sobkowic, Wojciech Kosmala, Tomasz Witkowski, Piotr Gosciniak, Alessandro Salustri, Hugo Rodriguez-Zanella, Luis Ignacio Martin Leal, Alexandra Nikolic, Suzana Gligorova, Madalina-Loredana Urluescu, Maria Fiorino, Giuseppina Novo, Tamara Preradovic-Kovacevic, Miodrag Ostojic, Branko Beleslin, Bruno Villari, Michele De Nes, Marco Paterni, Clara Carpeggiani, on behalf of Stress Echo 2020 study group of the Italian Society of Echocardiography and Cardiovascular Imaging (SIECVI), Eugenio Picano, Maria Grazia Andreassi, Clara Carpeggiani, Michele De Nes, Marco Paterni, Lorenza Pratali, Quirino Ciampi, Bruno Villari, Eduardo Bossone, Rodolfo Citro, Francesco Ferrara, Paolo Colonna, Marco Fabio Costantino, Lauro Cortigiani, Antonello D’Andrea, Claudio Dodi, Nicola Gaibazzi, Maurizio Galderisi, Andrea Barbieri, Ines Monte, Fabio Mori, Iacopo Olivotto, Barbara Reisenhofer, Federica Re, Fausto Rigo, Maria Chiara Scali, Sergio Severino, Paolo Trambaiolo, Miguel Amor, Jorge Lowenstein, Pablo Martin Merlo, Clarissa Borguezan Daros, José Luis de Castro, Silva Pretto, Marcelo H. Miglioranza, Marco A. R. Torres, Daniel Quesada Chaves, Melissa Rodriguez Israel, Iana Simova, Albert Varga, Gergely Agoston, Attila Palinkas, Jelena Čelutkienė, Jaroslaw D. Kasprzak, Karina Wierzbowska-Drabik, Ana Djordjevic-Dikic, Branko Beleslin, Milica Dekleva, Aleksandar N. Neskovic, Ivan Stankovic, Angela Zagatina, Giovanni di Salvo, Julio E. Perez, Ana Camarozano, Anca Corciu, Alla Boshcenko, Fabio Lattanzi, Carlos Cotrim, Paula Fazendas, Maciej Haberka, Bozena Sobkowicz, Wojciech Kosmala, Tomasz Witkowski, Piotr Gosciniak, Alessandro Salustri, Hugo Rodriguez Zanella, Alexandra Nikolic, Suzana Gligorova, Madalina-Loredana Urluescu, Maria Fiorino, Giuseppina Novo, Tamara Preradovic-Kovacevic, Miodrag Ostojic, Dario Gregori

**Affiliations:** 1 CNR, Institute of Clinical Physiology, Biomedicine Department, Pisa, Italy; 2 Cardiology Division, Fatebenefratelli Hospital, Benevento, Italy; 3 Cardiology Department and Echocardiography Lab, University Hospital “San Giovanni di Dio e Ruggi d’Aragona”, Salerno, Italy; 4 Cardiology Hospital, Policlinico of Bari, Bari, Italy; 5 Cardiology Department, San Carlo Hospital, Potenza, Italy; 6 Cardiology Department, San Luca Hospital, Lucca, Italy; 7 Cardiology Department, Echocardiography Lab, Monaldi Hospital, Second University of Naples, Naples, Italy; 8 Casa di Cura Figlie di San Camillo, Cremona, Italy; 9 Cardiology Department, Parma University Hospital, Parma, Italy; 10 Department of Advanced Biomedical Sciences, Federico II University Hospital, Naples, Italy; 11 Cardiology Department, Modena University Hospital, Modena, Italy; 12 Cardio-Thorax-Vascular Department, Echocardiography lab, Policlinico Vittorio Emanuele, University of Catania, Catania, Italy; 13 Cardiology Department, Careggi Hospital, Florence, Italy; 14 Cardiology Division, Pontedera-Volterra Hospital, ASL Toscana 3 Nord-Ovest, Florence, Italy; 15 Cardiology Department, San Camillo-Forlanini Hospital, Rome, Italy; 16 Cardiology Department, Ospedale dell’Angelo Mestre-Venice, Venice, Italy; 17 Cardiology Department, Nottola Hospital, Siena, Italy; 18 Cardiology Department, Ospedale santa Maria Incoronata dell’Olmo, cava de’ Tirreni, Salerno, Italy; 19 Department of Cardiology, Sandro Pertini Hospital, Rome, Italy; 20 Cardiology Department, Ramos Mejia Hospital, Buenos Aires, Argentina; 21 Cardiodiagnosticos, Investigaciones Medicas, Buenos Aires, Argentina; 22 Cardiology Division, Hospital San José, Criciuma, Brasília, Brazil; 23 Hospital Sao Vicente de Paulo e Hospital de Cidade, Passo Fundo, Brazil; 24 Cardiology Institute of Rio Grande do Sul, Porto Alegre, Brazil; 25 Hospital de Clinicas de Porto Alegre - Universidade Federal do Rio Grande do Sul, Porto Alegre, Brazil; 26 Hospital San Vicente de Paul, Heredia, Costa Rica; 27 Acibadem City Clinic Cardiovascular Center, University Hospital, Sofia, Bulgaria; 28 Institute of Family Medicine, University of Szeged, Szeged, Hungary; 29 Centre of Cardiology and Angiology, Vilnius University Hospital Santaros Klinikos, Faculty of Medicine, Vilnius University, State Research Institute for Innovative Medicine, Vilnius, Lithuania; 30 Chair of Cardiology, Bieganski Hospital, Medical University, Lodz, Poland; 31 Cardiology Clinic, Clinical Center of Serbia, Medical School, University of Belgrade, Belgrade, Serbia; 32 Clinical Hospital Zvezdara Belgrade, Belgrade, Serbia; 33 Department of Cardiology, Clinical Hospital Center Zemun, Faculty of Medicine, University of Belgrade, Belgrade, Serbia; 34 Cardiology Department, University Hospital, Saint Petersburg, Russian Federation; 35 Pediatric Cardiology Department, Brompton Hospital, London, UK; 36 Washington University School of Medicine, Barnes-Jewish Hospital, St. Louis, MO USA; 37 Hospital de Clinicas UFPR, Medicine Department, Federal University of Paranà, Curitiba, Brazil; 38 Department of Cardiology, IRCCS Policlinico San Donato Clinic, Milan, Italy; 39 Cardiology Research Institute, Tomsk National Research Medical Center of Russian Academy of Sciences, Tomsk, Russia; 40 Cardiothoracic Department, University of Pisa, Pisa, Italy; 41 Heart Center, Hospital da Cruz Vermelha, Lisbon and Medical School of University of Algarve, Faro, Portugal; 42 Cardiology Department, Hospital Garcia de Orta, Almada, Portugal; 43 Department of Cardiology, School of Health Sciences, Medical University of Silesia, Katowice, Poland; 44 Department of Cardiology, Medical University of Białystok, Białystok, Poland; 45 Department of Cardiology, Wroclaw Medical University, Wroclaw, Poland; 46 Department of Cardiology, Provincial Hospital, Szczecin, Poland; 47 Hamad Medical Corporation, Heart Hospital, Doha, Qatar; 48 Instituto Nacional de Cardiologia Ignacio Chavez, Mexico City, Mexico; 49 Institute for Cardiovascular Diseases, Dedinje, Belgrade, Italy; 50 Cardiology Division Ospedale Casilino, Rome, Italy; 51 Cardiology Department, County Hospital Sibiu, Invasive and Non-Invasive Center for Cardiac and Vascular Pathology in Adults - CVASIC Sibiu, Faculty of Medicine, Sibiu, Romania; 52 Cardiology Division Ospedale Civico Di Cristina Benfratelli, Palermo, Italy; 53 Cardiology Division, University Hospital, Palermo, Italy; 54 University Clinical Center, Banja Luka, Republic of Srpska Bosnia and Herzegovina

**Keywords:** Certification, Lung comets, Quality control, Stress echocardiography, Wall motion

## Abstract

**Background:**

The effectiveness trial “Stress echo (SE) 2020” evaluates novel applications of SE in and beyond coronary artery disease. The core protocol also includes 4-site simplified scan of B-lines by lung ultrasound, useful to assess pulmonary congestion.

**Purpose:**

To provide web-based upstream quality control and harmonization of B-lines reading criteria.

**Methods:**

60 readers (all previously accredited for regional wall motion, 53 B-lines naive) from 52 centers of 16 countries of SE 2020 network read a set of 20 lung ultrasound video-clips selected by the Pisa lab serving as reference standard, after taking an obligatory web-based learning 2-h module (http://se2020.altervista.org). Each test clip was scored for B-lines from 0 (black lung, A-lines, no B-lines) to 10 (white lung, coalescing B-lines). The diagnostic gold standard was the concordant assessment of two experienced readers of the Pisa lab. The answer of the reader was considered correct if concordant with reference standard reading ±1 (for instance, reference standard reading of 5 B-lines; correct answer 4, 5, or 6). The a priori determined pass threshold was 18/20 (≥ 90%) with R value (intra-class correlation coefficient) between reference standard and recruiting center) > 0.90. Inter-observer agreement was assessed with intra-class correlation coefficient statistics.

**Results:**

All 60 readers were successfully accredited: 26 (43%) on first, 24 (40%) on second, and 10 (17%) on third attempt. The average diagnostic accuracy of the 60 accredited readers was 95%, with R value of 0.95 compared to reference standard reading. The 53 B-lines naive scored similarly to the 7 B-lines expert on first attempt (90 versus 95%, *p* = NS). Compared to the step-1 of quality control for regional wall motion abnormalities, the mean reading time per attempt was shorter (17 ± 3 vs 29 ± 12 min, *p* < .01), the first attempt success rate was higher (43 vs 28%, *p* < 0.01), and the drop-out of readers smaller (0 vs 28%, *p* < .01).

**Conclusions:**

Web-based learning is highly effective for teaching and harmonizing B-lines reading. Echocardiographers without previous experience with B-lines learn quickly.

## Background

Stress echocardiography (SE) has some advantages over competing imaging techniques, including low cost, portability, radiation-free nature and versatility. Its major limitation is the dependence upon operator’s expertise, which may impact on the quality and consistency of diagnostic results [1, 2]. This limitation is magnified when the technique is used for scientific purposes in a multi-center trial such as Stress Echo 2020 (SE2020) study, designed to provide effectiveness data in 10,000 patients from > 100 laboratories in a variety of conditions ranging from coronary artery disease to heart failure (with preserved or depressed ejection fraction), hypertrophic cardiomyopathy, repaired congenital heart disease, valvular heart disease and extreme physiology [3]. To achieve harmonization, one possible approach is the use of the core lab which analyses centrally images sent from all recruiting sites. This approach is typically the preferred choice in a clinical trial and minimizes the sources of measurement variability [4, 5]. The core lab option was discarded in SE 2020 for two reasons. First, it was too costly and logistically demanding. Second, it would provide efficacy data under ideal conditions, but our aim was to obtain effectiveness data realistically generated when the technique is deployed in the clinical arena, populated by real patients, real doctors and real problems [6]. A feasible approach to ensure consistency in data acquisition and interpretation in this challenging setting is to develop an upstream reading quality control for prospective centers willing to enter the study [7, 8]. In SE2020, this approach has already been implemented for regional wall motion abnormalities (RWMA), which remains the diagnostic cornerstone of SE [9]. However, a separate quality control needs to be performed for other aspects of contemporary SE practice, such as B-lines obtained with lung ultrasound (LUS) [10]. Also known as ultrasound lung comets, B-lines are a sign of accumulation of extra-vascular lung water [11] and can acutely increase during stress [12–14]. Their presence and/ or increase during stress places the patient in a higher risk subset for any level of RWMA [13] and indicates that dyspnea is linked to acute backward heart failure [15]. B-lines assessment must be properly standardized and quality-controlled prior to dissemination and use for clinical and scientific purposes. The present report was part of the larger SE2020 study and focuses on the educational aspects of LUS-SE, describing the results of the upstream quality control and harmonization of B-lines reading criteria across 52 SE2020 centers.

## Methods

**Fig. 1:**
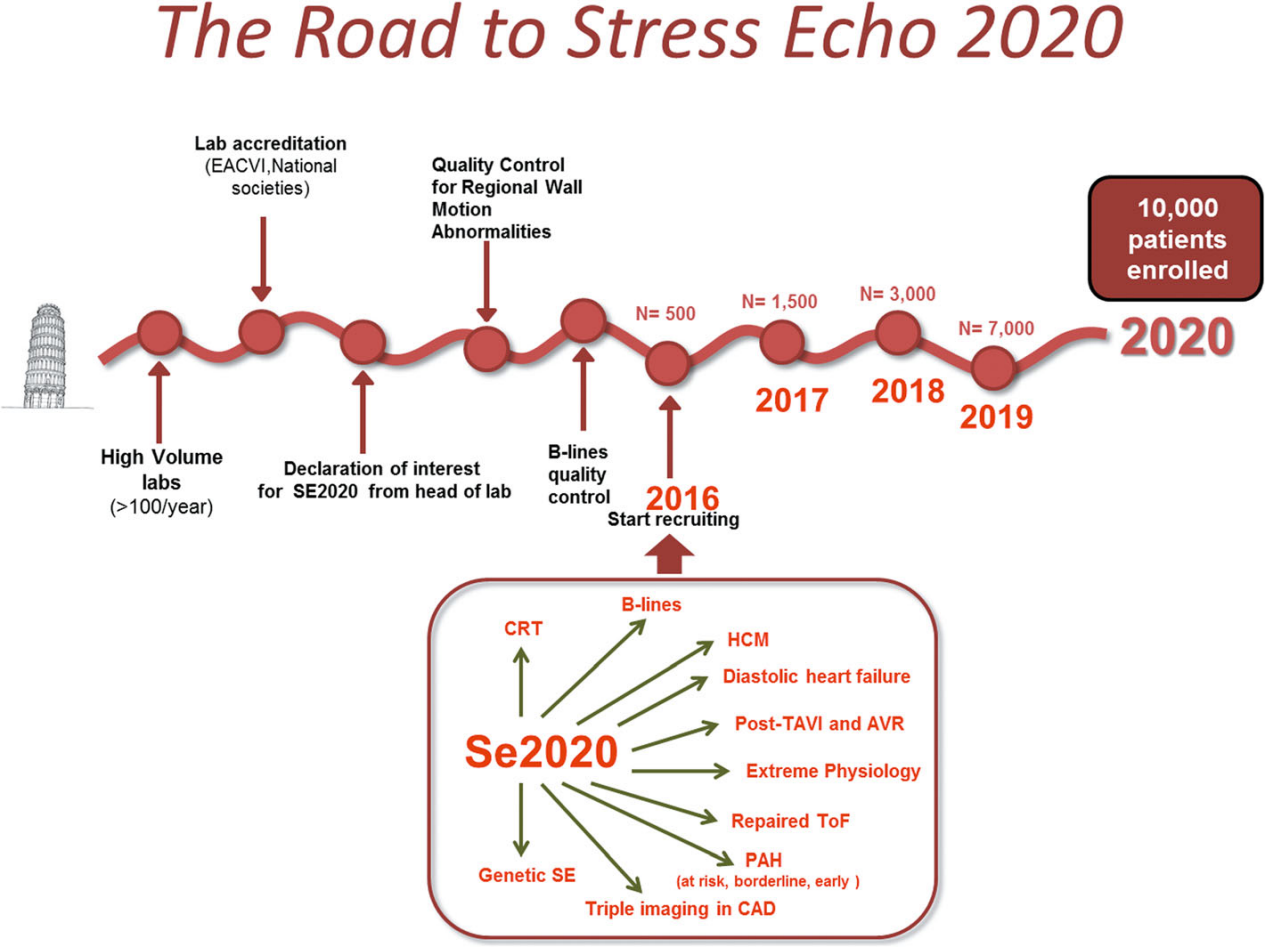
The road to accreditation for the aspiring recruiting centers. After the first essential step of RWMA, the reader completes the second step (B-lines) and starts enrolling with dual imaging (RWMA and B-lines)

The Pisa lab coordinated the quality control assessment for B-lines of all investigators who expressed their intention to participate in the study (Fig. 1). The coordinating center was in the National Research Council, Institute of Clinical Physiology in Pisa, Italy. The candidate centers included 52 centers (each with at least one certified reader) from 16 countries (Argentina, Brazil, Bulgaria, Costa Rica, Hungary, Italy, Lithuania, Mexico, Poland, Portugal, Romania, Qatar, Russia, Serbia, UK, USA). The selection criterion was that all readers had already passed the quality control for RWMA reading (step 1 in the “Road to SE 2020”). The B-lines reading was the step 2 in the “Road to SE 2020”. The complete list of participants in the SE2020 consortium (as per January 20th, 2018) is reported in the Appendix. The study protocol was reviewed and approved by the institutional ethics committee as a part of the SE 2020 study (1487-CE Lazio-1, July 20, 2016). The study was funded with institutional funding of the Italian National Research Council and with travel grants of the Italian Society of Echocardiography and Cardiovascular Imaging with dedicated sessions during national meetings. No fort from industry was asked for or received.

An obligatory web-based educational platform was developed to facilitate the training process. Participating cardiologists were invited by email to join the platform, which was protected by user-specific passwords. The platform includes files and videos with detailed instructions on how to start the training and allows downloading and uploading of external files. The sequence of the certification process and web-based learning has already been detailed and follows the same template used for RWMA [9]. We decided to have this platform mandatory and not optional as in the step-1 for RWMA, since in case of B-lines the technique is relatively young and recent advances in acquisition (with 4-site scan mode) and reporting were adopted in the SE2020 platform [16].

### Study population of readers

Sixty readers from 52 different centers initially asked to enter the SE2020 study, had passed the RWMA test for quality control and therefore were allowed to enter the step-2 of SE2020.

All participants were clinical cardiologists and expert echocardiographers with ongoing high volume (> 100 tests per year) SE activity and the years of experience in SE ranged from 5 to 31 years (mean value 18 years). All were certified by national and/or international societies .

### Lung ultrasound acquisition

**Fig. 2:**
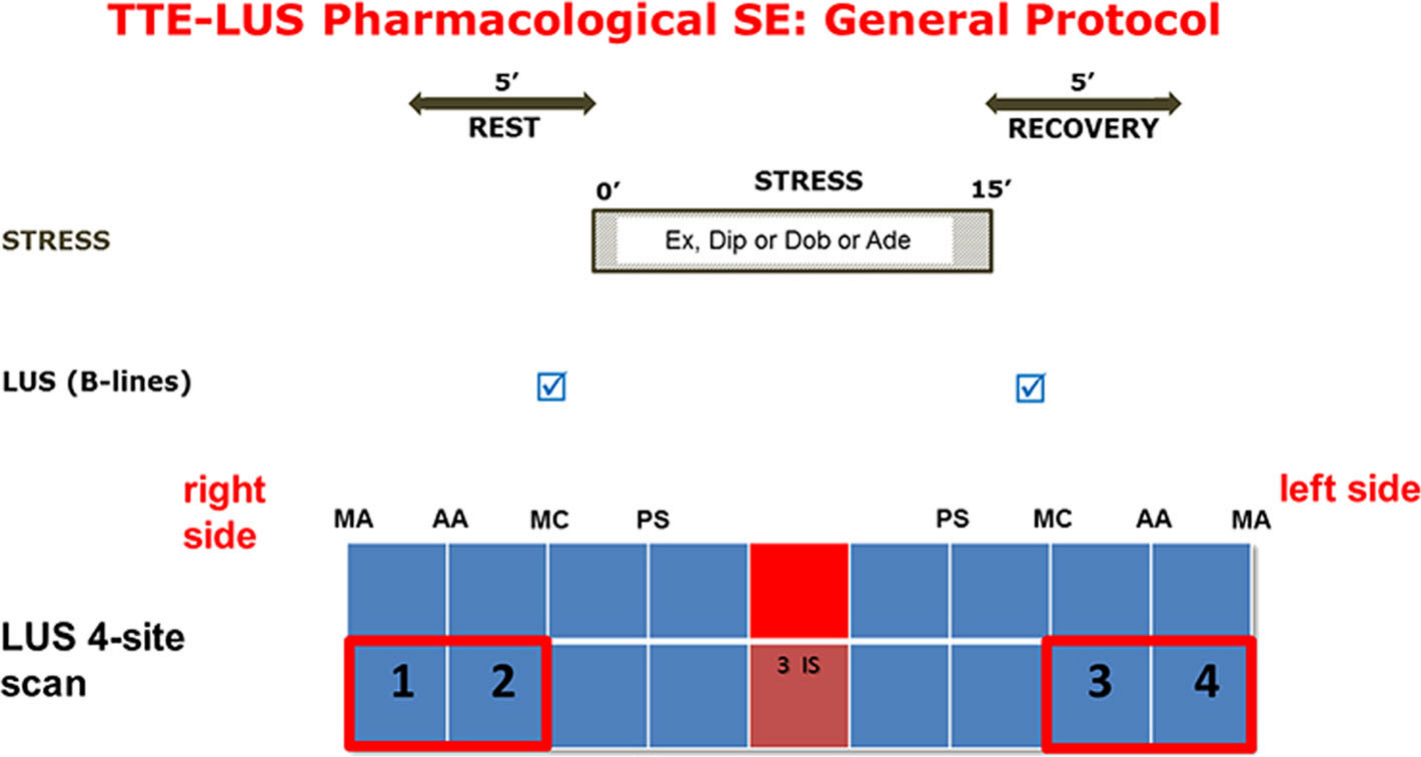
The Stress-LUS general protocol. LUS for B-lines are assessed at baseline and at the end of stress, after the acquisition for RWMA. The adopted protocol is the 4-site simplified scan

To acquire lung ultrasound (LUS) images adopted for quality control test, we used commercially available ultrasound machines (IE 33, Philips, Medical Systems, Andover, Massachusetts, USA with a 2.5–3.5 MHz phased-array sector scan probe; Vivid E9, GE Healthcare, USA, manufactured in Horten, Norway, equipped or standard M5S transducer with second harmonic technology; Mylab Eight platform Esaote, Genova, Italy). The depth was adjusted according to the body habitus of the patient, with thin patients requiring less depth and obese patients needing greater depth to visualize the pleural line. A B-line was defined with 4 constant criteria: vertical, laser-like, hyperechoic reverberation; arises from the pleural line extending to the bottom of the screen without fading; moves synchronously with lung sliding; and erases the A-lines, which are a part of the normal lung pattern as a horizontal, multiple reverberation artefact, equidistant from one another below the pleura, at exact multiples of the transducer-pleural line distance [17]. Detailed description of the scanning procedure and scanning sites is also available in a 2-min movie from our laboratory on YouTube (The incredible ULCs – ultrasound lung comets. Available at http://www.youtube.com/watch?v=7y_hUFBHStM. Accessed: July 10, 2018). LUS scanning was performed with the cardiac probe in the supine position at rest and soon after stress (with the patient again resuming the supine position). The 4-site simplified scan of the lung was used [16]. We analyzed the anterior and lateral hemithoraces, scanning along the anterior axillary (AA) and midaxillary (MA) lines on the third intercostal space (Fig. 2).

### Web-based learning module

**Fig. 3:**
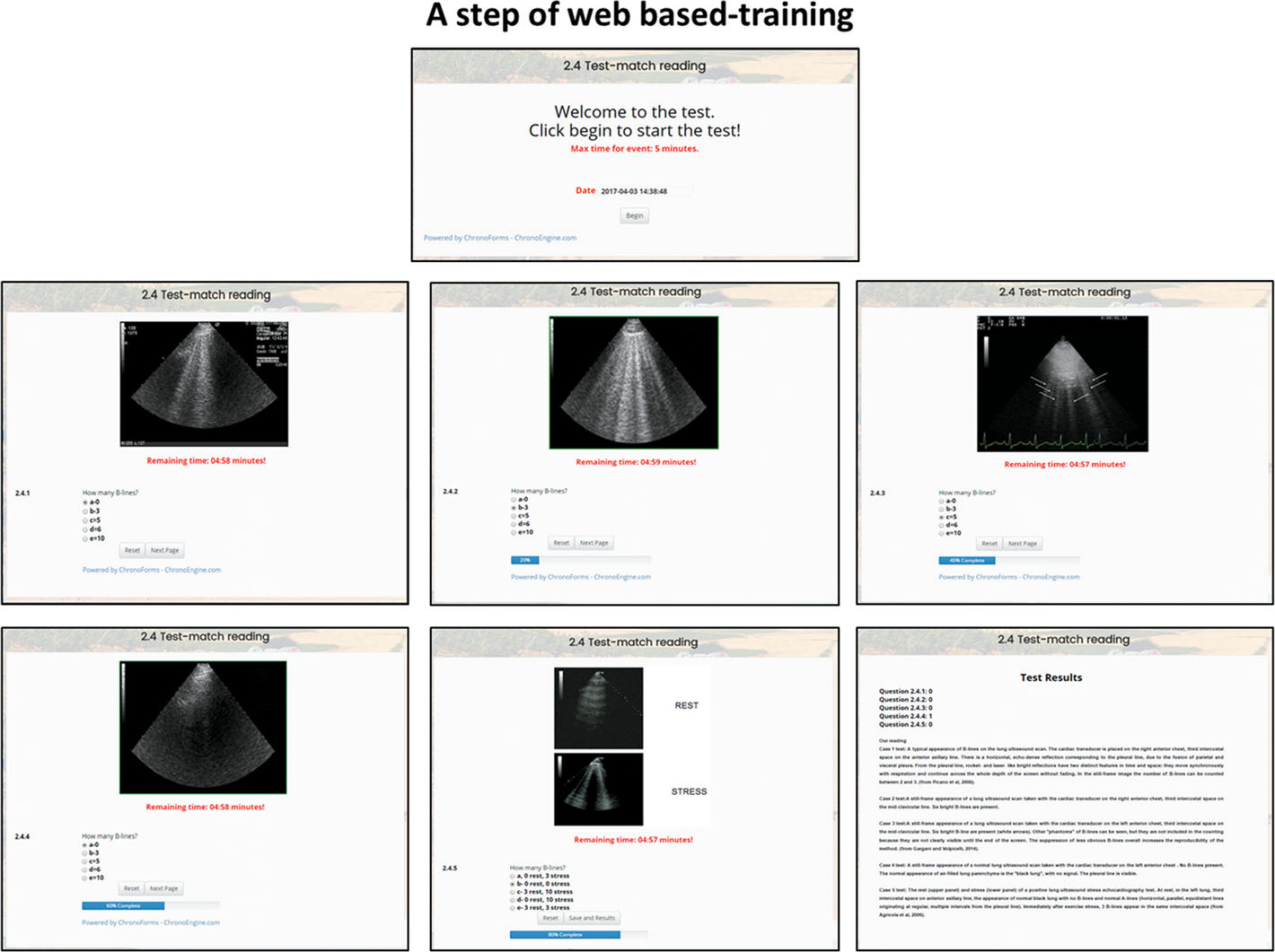
The screenshot of the test-match step during B-lines quality control. There are 5 still frames (or videos) with B-lines and the trainee has to choose among 5 possible answers, ranging from 0 (left lower panel) to 9 (upper middle panel)

The 2-h web-based training module (http://se2020.altervista.org) consisted of five sequential learning blocks: **a-** Selected readings of 3 recent review or original articles summarizing the evidences supporting the use of B-lines during stress and the adopted scan technique and scoring criteria [8, 10, 13]; **b**- A power-point file of 25 slides summarizing key points and specific literature supporting the proposed reading policy illustrating tips and tricks highlighting the most frequent problems in B-lines interpretation with special focus on the technicalities of the 4-site simplified scanning technique; **c**- A theory self-assessment test with five questions with four answers each (only one correct) preliminary to video-clip reading; **d**- Short (< 15 s) video-clips of examinations with the same format of official test reading, with 5 min per reading with countdown clock, and one possible answer (from 0 to 10) for each video-clip (Fig. 3).

An expert trainer (QC or MCS) remained available to all readers for e-mail or phone contact to provide assistance with any issue concerning the training.

At all times there was the possibility of face-to-face discussion (via Skype) to address issues requiring special clarification with the principal investigator. After completing the web-based module the reader could take the test (maximum three attempts). After each attempt, the sequence of videos was mixed.

### Reading sessions and pass threshold

We selected 20 cases of 10 patients (with rest and stress images) in which the presence and number of B-lines was documented by unanimous decision of 2 experienced observers (EP and QC). The privacy of patients during acquisition, storage, and transmission of the SE study was protected. All images were anonymized, and the identity of patients or the study condition (rest or stress) was not disclosed at any time to the readers. Each SE study was structured in a single video-clip of 10–15 s, with either resting or stress images. Each test clip was scored from 0 (black lung, A-lines, no B-lines) to 10 (white lung with coalescing B-lines). The diagnostic gold standard was the reading of Pisa lab. The answer of the reader was considered correct if concordant with reference standard reading ±1 (for instance, reference standard reading of 5 B-lines; correct answer 4, 5, or 6). The a priori determined pass threshold was 18/20 (≥ 90%) with R value of intra-class correlation coefficient > .90.

The LUS images were selected to represent the garden variety of stress testing modes, responses, results and image quality. They came from six different laboratories (Benevento, Lucca, Pisa, Porto Alegre, Rome, St Petersburg) in three countries (Brasil, Italy, Russian Federation), and showed the full spectrum of responses (from 0, *n* = 7; to 10, *n* = 1). All images were considered readable, with quality ranging from average-to-good (*n* = 16) to excellent (*n* = 4) in the assessment of the reference standard reading. The stress employed was exercise in 17 subjects, high dose accelerated dipyridamole (0.84 mg/kg over 6 min) in 2 and dobutamine (40 mcg/kg/min) in 1. The projection selected was the third intercostal space between left mid-axillary and anterior axillary lines in 4; third intercostal space between right mid-axillary and anterior axillary lines in 4; third intercostal space between right anterior-axillary and mid-clavicular lines in 4; third intercostal space between left anterior-axillary and mid-clavicular lines in 8.

### After the pass or fail response

The response was pass (≥ 90% accuracy) or fail. With pass, the reader received a certificate of accreditation and could start recruiting with a written informed consent signed by each patient and after clearance by the local ethical committee. With fail, the unsuccessful reader could retake the test after 1 month. After the second fail, the reader could undergo training in a recommended center and try again after 1 year.

### Statistical analysis

Each reader was evaluated against the gold standard of reference standard reading for assessment of individual accuracy (in %). The intra-class correlation coefficient was calculated, for each reader, in the whole series of 20 paired measurements made by the peripheral reader and the reference reader. Intra-observer agreement was tested in 20 peripheral readers who volunteered to repeat the measurement session after at least 3 months from the first reading. A *p* value < 0.05 was considered significant.

## Results

**Fig. 4:**
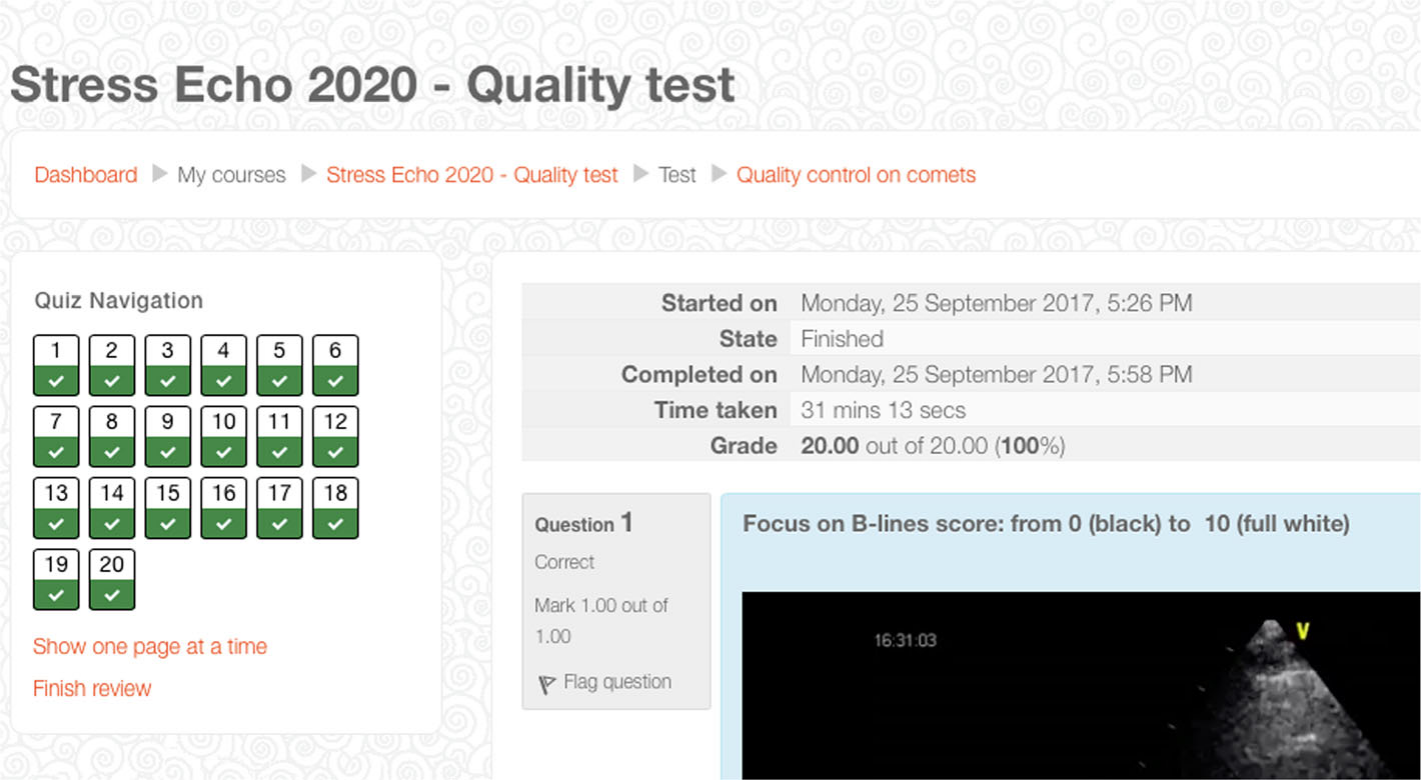
The test results of a reader passed with full marks (20/20)

Of the initial 60 readers who started, 53 were B-lines naive (without previous exposure to B-lines). All 60 readers were successfully accredited (Fig. 4): 26 (43%) on first, 24 (40%) on second, and 10 (17%) on third attempt. The 53 B-lines naive scored similarly to the 7 B-lines expert on first attempt (90 versus 95%, *p* = NS). Compared to the step-1 of quality control for regional wall motion abnormalities [6], the mean reading time per attempt was shorter (17 ± 3 vs 29 ± 12 min, *p* < .01), the first attempt success rate was higher (43 vs 28%, *p* < 0.01), and the drop-out of readers smaller (0 vs 28%, *p* < .01). The average diagnostic accuracy of the 60 accredited readers was 95%. Considering the final attempt of the 60 readers, the Spearman correlation coefficient between the expert reference reading and the reading of each peripheral reader was very high (*R* = 0.95, *p* < .0001). In the 20 peripheral readers who repeated the test a second time at least 3 months after accreditation, the Spearman correlation coefficient was also very high (*R* = 0.97, *p* < .0001).

## Discussion

A user-friendly web-based learning is highly effective for training B-lines also for echocardiographers without previous exposure to B-lines. After a limited learning effort, the accuracy of B-lines reading is comparable between very experienced and freshly trained readers. B-lines with 4-site simplified scan of the lung has a very high success rate in acquisition and analysis. It has been embedded as an integral part of dual imaging SE adopted as the core protocol in SE2020 for all forms of physical and pharmacological stress for all patients, from coronary artery disease to heart failure.

### Comparison with previous studies

The American College of Chest Physicians has defined the knowledge and technical elements required for competence in lung ultrasound [18]. There have been a number of prior lung ultrasound education papers, showing that a limited training of a few hours can improve the capability of execution and interpretation of LUS even in medical students without previous exposure to ultrasound [19, 20]. In the present study we are dealing with a specific and limited aspect of LUS of special interest for cardiologists, i.e. the detection of B-lines. There is a lack of a specific training and certification pathway in cardiology, and as a result training and performance of LUS varies widely among different institutions. An approach similar to the one adopted in the present study was developed in Pisa for centers recruiting in the LUST study [21]. However, this study differs from the previous one under some aspects: first, it was focused on LUS-SE, not on resting LUS; the adopted scan scheme was the simplified 4-site scan, easier to do, to teach and to learn than the previously adopted 28-site scan; and the quality control procedures required some prior reading and slide presentation to facilitate a standardized learning [9].

Our findings are consistent with a large body of literature showing that stable web applications are increasingly used for improving medical image interpretation skills regardless of time and space and without the need for expensive imaging equipment or a patient to scan [22]. With the adopted web-based approach, the educational path is standardized, shared, and - after validation and refinement - prospectively available in open source, and exploitable for scientific purposes and clinical education. The use of enabling technologies makes the accreditation process faster, smoother and cheaper, and coupled with the open-source platform grants an unprecedented opportunity for continuing education, also fostered by endorsement and governance by the scientific society supporting the study.

### Study limitations

We focused on the assessment of B-lines, which is a particularly simple aspect of LUS diagnosis [10, 11]. Similar harmonization and accreditation issues are present for other aspects of SE diagnosis. Separate and parallel training modules are currently under construction within the framework of “SE 2020” to cover the entire spectrum of key aspects of SE diagnosis, from coronary flow velocity reserve to left ventricular volumes and pulmonary hemodynamics [3].

A key aspect in the evaluation of SE results is the adoption of an undisputed diagnostic “gold standard”. The lack of a universally acceptable gold standard makes the assessment of reading performance difficult. From the library of images arriving from all the world and stored in our data bank, we selected cases meeting the conditions of unanimous reading of the two most experienced readers from the reference lab. This is a far from perfect gold standard, yet a reasonable, and perhaps the only possible, one.

We restricted our validation phase to participants in the SE2020 study, who had a substantial reading experience and certification in RWMA as a prerequisite. This reader pool may have been especially knowledgeable and motivated, thereby justifying the excellent learning results. However, 53 of them were B-lines naive, and therefore probably the selection criteria of our readers did not affect the generalizability of results.

We adopted a simplified 4-site scan for acquisition of B-lines at rest and during stress. This approach introduces a substantial abbreviation compared to other protocols such as the 28-region scan originally adopted in the Pisa laboratory in the first application of LUS in heart failure patients [23] and also recommended by an international consensus in 2012 [24]. Over the years, simplified 8-zone and 4-zone lung imaging protocols were proposed [25, 26], with comparable information between the 2 protocols as shown by Platz et al. [25]. Scali et al. showed that the simplified 4-site scan allows to complete the assessment of B-lines in 20 s (instead of the 3 min required by the 28- region scan). There is a linear, close correlation between the 28-site and the 4-site B-lines score [16]. Therefore, there is no significant loss of information when going from 28- to 4-site scan, but a substantial simplification and time saving, vital for SE imaging, when there are so many things to see and so little time available.

### Clinical implications

B-lines are a useful adjunct to mainstream SE based on RWMA [27, 28], but its impact may be limited by the relatively few centers currently using it in their routine SE practice, and the lack of standardization in acquisition, scoring and reporting [29]. After a web-based module and certification, the approach is better harmonized and the accumulation of clinical practice also allows the rapid growth of scientifically unique data. To achieve this goal, simplification is essential, and the 4-site simplified scan is ideal for LUS rest and stress testing.

However, the SE technique does not tolerate improvisation, and an accurate standardization of terminology, standards of execution, and interpretation criteria is required before a center is allowed to enter its experience in the common data bank. Similarly to what has been said for meta-analysis [30], multicenter SE studies are like a bouillabaisse: no matter how much seafood (or recruiting centers) is added, one tainted fish (an unreliable center generating inconsistent reading) will spoil the pot.

## Conclusion

Web-based learning is highly effective for teaching and harmonizing B-lines reading, with an enormous saving of time and resources versus the conventional hands-on approach of teaching and learning ultrasound techniques. Echocardiographers without previous experience with B-lines learn quickly.

## Abbreviations

CAD: Coronary artery disease
LUS: Lung Ultrasound
RWMA: Regional wall motion abnormalities
SE: Stress echocardiography
TE: Transthoracic echocardiography
